# Dysmagnesemia Is the Most Common Disturbance of the Calcium–Magnesium–Phosphorous Balance among Older Hospitalized People in Warsaw

**DOI:** 10.3390/nu13103395

**Published:** 2021-09-27

**Authors:** Justyna Malinowska, Milena Małecka-Giełdowska, Olga Ciepiela

**Affiliations:** 1 Department of Laboratory Medicine, Medical University of Warsaw, 02-091 Warsaw, Poland; justyna.malinowska@wum.edu.pl; 2 Doctoral School at Medical University of Warsaw, 02-091 Warsaw, Poland; milena.malecka@wum.edu.pl; 3 Central Laboratory, Central Teaching Hospital of University Clinical Center of Medical University of Warsaw, 02-091 Warsaw, Poland

**Keywords:** calcium, elderly, magnesium, phosphorous, vitamin D3

## Abstract

The elderly are at great risk of developing life-threatening disturbances in calcium–magnesium–phosphate homeostasis because of comorbidities, long-term medication use, and dietary deficiencies, but it is still not known how often they occur in this group of patients. This study aimed to assess the prevalence of these disturbances in a group of hospitalized patients over 65 years of age according to age and sex. The study was conducted between January 2018 and September 2020 at the Central Clinical Hospital in Warsaw. A total of 66,450 calcium, magnesium, phosphate, and vitamin D concentration results were included in the analysis. Dysmagnesemia was present in 33% of the calcium results, dyscalcemia, dysphosphatemia, and dysvitaminosis D—in 23.5%, 26%, and 70% of the results, respectively. The magnesium concentration was found to be age-dependent, and older people were found to be at higher risk of developing abnormal magnesium concentrations (*p* < 0.001). Sex influenced the occurrence of abnormal magnesium (*p* < 0.001), vitamin D (*p* < 0.001), and calcium (*p* < 0.00001) concentrations, with hypercalcemia and hypervitaminosis D disorders being significantly more common in women (*p* < 0.0001). In conclusion, disorders of the calcium–magnesium–phosphate metabolism are common in hospitalized patients over 65 years of age, and the concentrations of these substances should be routinely monitored in this group.

## 1. Introduction

With the increasing life expectancy and the demographic changes seen in Europe, the elderly are expected to comprise an increasing proportion of the population [1]. In 2018, people over 65 accounted for 19.7% of Europe’s population. According to the Eurostat projections for 2050, 28.5% of Europe’s population will be over 65 [2]. As a result, there will be an increase in the number of patients suffering from illnesses that are typical of old age [3].

Age over 65 is associated with an increased incidence of certain health problems. They include abnormal serum calcium, magnesium, and phosphate concentrations, which are common in hospitalized patients and are associated with an increased risk of death [4]. Factors predisposing to their occurrence include age, malnutrition, diabetes, chronic kidney disease, and the use of diuretics [5]. Although the prevalence of disturbances in calcium–magnesium–phosphate homeostasis in people over 65 is not yet well-documented, they play a role in the pathogenesis of other diseases such as cardiovascular disease [6,7,8], type 2 diabetes [6,9], osteoporosis [10,11], frailty syndrome [3], and sarcopenia [12], which often affect people of this age. An additional factor that influences calcium–magnesium–phosphate homeostasis and bone health is the concentration of vitamin D. Older people are more prone to deficiency of this vitamin because its dermal synthesis is reduced. This is because of less frequent exposure to sunlight and a decreased amount of vitamin D precursors in the skin. In addition, the diets of older people are often less varied and poorer in natural sources of vitamin D [13].

This study aimed to assess the prevalence of disturbances in calcium–magnesium–phosphate homeostasis in hospitalized people over 65 years of age due to their serious health consequences and involvement in the pathogenesis of diseases that commonly affect older adults.

## 2. Materials and Methods

**Figure 1:**
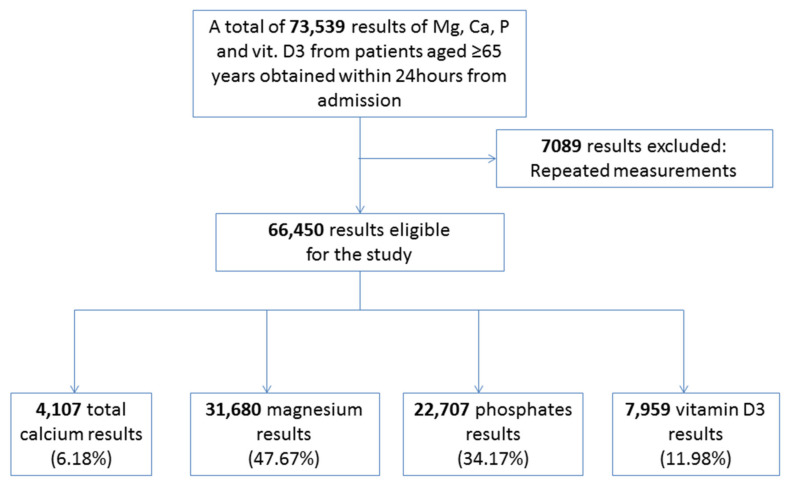
Enrolment scheme—study group selection. Mg, magnesium; Ca, calcium; P, phosphates; vit. D3, vitamin D3.

A retrospective analysis of serum calcium, magnesium, phosphate, and vitamin D concentrations was performed among the patients hospitalized at the Central Clinical Hospital of the Medical University of Warsaw between January 2018 and September 2020. A total of 73,539 results of patients aged 65 years and over were included in the study. Final analysis was performed using laboratory results from 66,450 hospitalized patients obtained from the laboratory information system (Figure 1). All the included values were obtained only at the beginning of hospitalization to avoid the negative impact of repeated measurements on the obtained results. This population-based study includes only raw laboratory data without any relationship to the clinic. The patients were hospitalized at the Departments of Diabetology, Cardiology, General Surgery, Endocrinology, Hematology and Oncology, Nephrology, Laryngology, Intensive Care, Gastroenterology Surgery, Vascular Surgery, Hepatic Surgery, Neurosurgery, Cardiac Surgery, Hypertensiology and Internal Medicine, Gastroenterology, Pneumology, Nephrology and Dialysis, and Hepatology (Table 1). The criteria for inclusion in the study were age 65 years or over and a performed serum calcium, albumin, magnesium, phosphate, or vitamin D test. Among the patients who had their serum calcium levels measured, we chose those who also had their albumin and vitamin D levels tested. We estimate that 29.5% of the patients had conditions that may have affected the results: diabetes, primary and secondary hyperparathyroidism, and chronic kidney disease. In addition, we estimate that 12.5% of the patients took drugs that may have affected the state of their calcium–magnesium–phosphate homeostasis and serum vitamin D concentrations. These drugs include diuretics, cyclosporine, tacrolimus, and platinum derivatives. The study group selection is shown in the diagram below. The extracted results were obtained within 24 h from admitting the patient.

Calcium, magnesium, phosphate, and vitamin D concentrations were tested upon admission. Peripheral blood was collected by venipuncture into tubes containing a clotting accelerator and allowed to clot for 30 min. The tubes were then centrifuged for 10 min at 1500× *g*. Total magnesium, calcium, and phosphates in serum were measured using the colorimetric method. The principle of the method consists in measuring the absorbance of the reaction product of the mineral with the working agent. Magnesium reacts with xylidyl blue, resulting in a violet diazonium salt; meanwhile, calcium reacts with Arsenazo III, which results in the creation of a blue complex, and phosphates react with ammonium molybdate, forming colored phosphomolybdate complexes. The absorbance of the reaction product is directly proportional to the serum concentration of the mineral. We defined reference values for magnesium based on data from our previous study [14]. Vitamin D concentrations were measured using the Roche Cobas assay, in which polyclonal antibodies bind specifically to human 25(OH)D during incubation. Then, the reaction mixture is transferred to the measurement chamber, where microparticles are attracted to the electrode surface by a magnet. The voltage applied to the electrode induces the electrochemiluminescence reaction and photon emission, which is measured with the help of a photomultiplier. The measurement range was 3–100 ng/mL (7.5–250 nmol/L), with intermediate precision (coefficient of variation (CV) ≤ 20%). The accuracy for the lower boundary of 25(OH)D was 1.08 ng/mL, with a CV of 7.7%; for the upper boundary—1.3 ng/mL, with a CV of 4.03%.

The analyzers used to take measurements were Cobas 702 (Hoffmann-La Roche AG, Basel, Switzerland), Cobas 8000 (Hoffmann-La Roche AG, Basel, Switzerland), Dimension 1 (Siemens Healthineers Germany), and Dimension 2 (Siemens Healthineers Germany). For calcium measured with the Dimension 1 and 2 analyzers, the measurement range was 5.0–15.0 mg/dl (1.25–3.75 mmol/L), and the precision was high (coefficient of variation (CV) of 4.9–5%). For magnesium measured with the Dimension 1 and 2 analyzers, the measurement range was 0.0–20.0 mg/dl (0.0–8.22 mmol/L), and the precision was high (coefficient of variation (CV) of 1.7–1.9%). For magnesium measured with Cobas 702, the measurement range was 0.10–2.0 mmol/L (0.243–4.86 mg/dL), and the precision was high (coefficient of variation (CV) of 0.7–1.3%). For phosphates measured with the Dimension 1 and 2 analyzers, the measurement range was 0.5–9.0 mg/dl (0.16–2.91 mmol/L), and the precision was high (coefficient of variation (CV) of 1.3–2.7%).

In this study, we analyzed serum magnesium, calcium, phosphate, and vitamin D concentrations, age, and sex. Since being elderly is a risk factor for hypoalbuminemia, for those subjects who had decreased albumin concentration, we calculated a corrected calcium concentration using the following equation: Ca corrected = Ca + 0.02 × (40 – Alb), where Ca is the total calcium in mmol/l and Alb is albumin (g/L). The research was conducted according to the rules of the Bioethical Committee of the Medical University of Warsaw, and all the data were anonymized.

Statistical analysis was performed using Microsoft Office Excel 2019 and Statsoft Statistica. The tests used were the Mann–Whitney U test (effect of sex and age on serum calcium, magnesium, phosphate, and vitamin D concentrations), the Kruskal–Wallis test (the difference in the mean mineral concentration between the three groups and the difference between the age groups), Mood’s median test (difference in the median vitamin D concentration between the three groups), and Fisher’s exact test to analyze the frequency of dyscalcemia and dysvitaminosis D for each sex. A nonparametric Spearman correlation was used to assess the correlation of age with serum calcium, magnesium, phosphate, and vitamin D concentrations. The Shapiro–Wilk and Kolmogorov–Smirnov tests were used to assess the normality of the distribution of the results. We considered a *p*-value < 0.05 as statistically significant.

## 3. Results

Calcium, magnesium, phosphate, and vitamin D test results of hospitalized patients aged 65 years and over were included in the study. For the basic information on the patients and the test results, see Table 2.

### 3.1. Calcium

The serum calcium concentration was tested in 4107 patients, among whom 1225 patients had hypoalbuminemia. In these cases, we calculated the corrected calcium concentration. Among these patients, there were 1643 (40%) men and 2464 (60%) women. The mean calcium concentration was 2.4 ± 0.2 mmol/L. Hypocalcemia (calcium < 2.25 mmol/L) was found in 760 patients (18.5%), of which 334 (44%) were men and 426 (56%) were women. The mean calcium concentration in the hypocalcemia group was 2.13 ± 0.12 mmol/L. Normocalcemia (calcium concentration of 2.25–2.75 mmol/L) was found in 3142 subjects (76.5%), among whom 1241 (39.5%) were men and 1901 (60.5%) were women. The patients with normocalcemia had a mean calcium concentration of 2.43 ± 0.11 mmol/L.

Hypercalcemia (calcium concentration > 2.75 mmol/L) was found in 205 patients (5%), of whom 59 (29%) were men and 146 (71%) were women (*p* < 0.0001). The mean calcium concentration in the hypercalcemia group was 2.95 ± 0.21 mmol/L.

**Figure 2:**
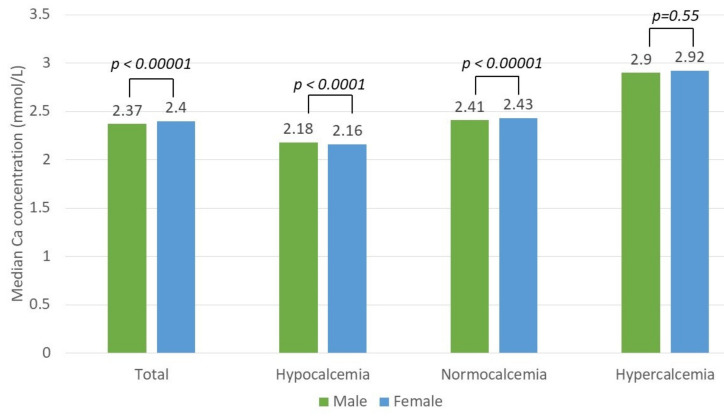
Difference in the median calcium (Ca) concentration between sexes in the total patient and hypo-, normo-, and hypercalcemia groups.

The difference between the median calcium concentrations in the three groups was statistically significant (*p* < 0.05) (Kruskal–Wallis test). The difference in the calcium concentration between the sexes was statistically significant in the total study group (*p* < 0.00001) and in the normocalcemia (*p* < 0.00001) and hypocalcemia (*p* < 0.0001) groups but not in the hypercalcemia group, *p* = 0.55 (Mann–Whitney U test) (Figure 2).

The correlation of age with serum calcium concentration was statistically significant but very weak (*p* < 0.001, *r* = −0.05). The median age differed statistically significantly between the hypo- and normocalcemia groups (*p* < 0.05) but not between the hypo- and hypercalcemia groups (*p* = 0.4) nor between the normo- and hypercalcemia groups (*p* = 1, Kruskal–Wallis test). Additionally, there was a statistically significant difference in the median calcium concentrations between the age groups (*p* < 0.05) (Table 3).

### 3.2. Magnesium

**Figure 3:**
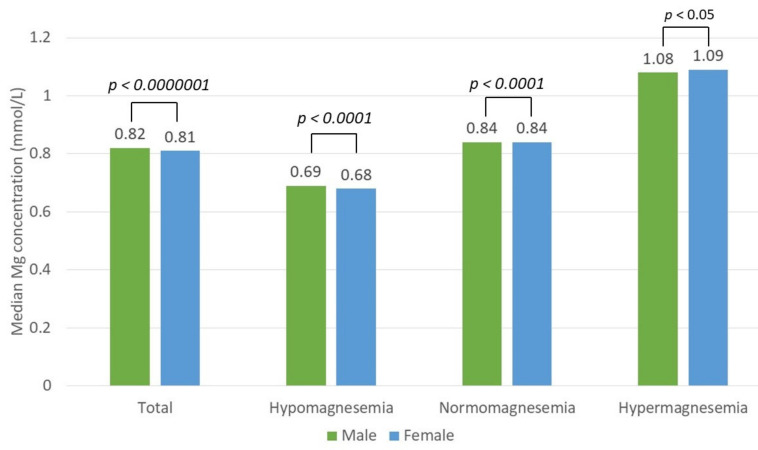
Median magnesium (Mg) concentration difference between sexes in the total patient and hypo-, normo-, and hypermagnesemia groups.

The serum magnesium concentration was tested in 31,680 patients, among whom 16,474 (52%) were men and 15,206 (48%) were women. The mean magnesium concentration was 0.82 ± 0.14 mmol/L. Hypomagnesemia (magnesium concentration < 0.75 mmol/L) was found in 8237 subjects (26%), of whom 4036 (49%) were men and 4201 (51%) were women. The mean magnesium concentration in the hypomagnesemia group was 0.66 ± 0.07 mmol/L. Normomagnesemia (magnesium concentration of 0.75–1.0 mmol/L) was found in 21,225 subjects (67%), among whom 11,037 (52%) were men and 10,188 (48%) were women. The patients with normomagnesemia had a mean magnesium concentration of 0.85 ± 0.06 mmol/L. Hypermagnesemia (magnesium concentration > 1.0 mmol/L) was found in 2218 subjects (7%), of whom 1264 (57%) were men and 954 (43%) were women. The mean magnesium concentration in the hypermagnesemia group was 1.13 ± 0.15 mmol/L. The median magnesium concentration differed significantly between the hypo-, normo-, and hypermagnesemia groups (*p* < 0.05, Kruskal–Wallis test). There was a statistically significant difference in magnesium concentration between sexes among the total patient (*p* < 0.0000001) and the hypo-, hyper-, and normomagnesemia groups (*p* < 0.0001, *p* < 0.05, and *p* < 0.0001 (Mann–Whitney U test), respectively) (Figure 3).

The correlation between age and the serum magnesium concentration was statistically significant, but very weak (*p* < 0.05, r = 0.012). However, the median age differed statistically significantly between the hypo-, normo-, and hypermagnesemia groups (*p* < 0.001), but there were no significant differences in the median magnesium concentration between the age groups (*p* = 0.07, Kruskal–Wallis test).

### 3.3. Phosphates

The phosphate concentration was tested in 22,707 patients, among whom 11,808 (52%) were men and 10,899 (48%) were women. The mean phosphate concentration was 1.19 ± 0.4 mmol/L. Hypophosphatemia (phosphate concentration < 0.8 mmol/L) was found in 2498 (11%) subjects, of whom 1399 (56%) were men and 1099 (44%) were women. The patients with hypophosphatemia had a mean phosphate concentration of 0.66 ± 0.12 mmol/L. Normophosphatemia (phosphate concentration of 0.8–1.5 mmol/L) was found in 16,803 (74%) patients, among whom 8738 (52%) were men and 8065 (48%) were women. The mean phosphate concentration in the normophosphatemia group was 1.12 ± 0.2 mmol/L.

Hyperphosphatemia (phosphate concentration > 1.5 mmol/L) was found in 3406 (15%) subjects, of whom 1839 (54%) were men and 1567 (46%) were women. The patients with hyperphosphatemia had a mean phosphate concentration of 1.9 ± 0.4 mmol/L.

The median phosphate concentration differed significantly between the three groups (*p* < 0.05, Kruskal–Wallis test).

**Figure 4:**
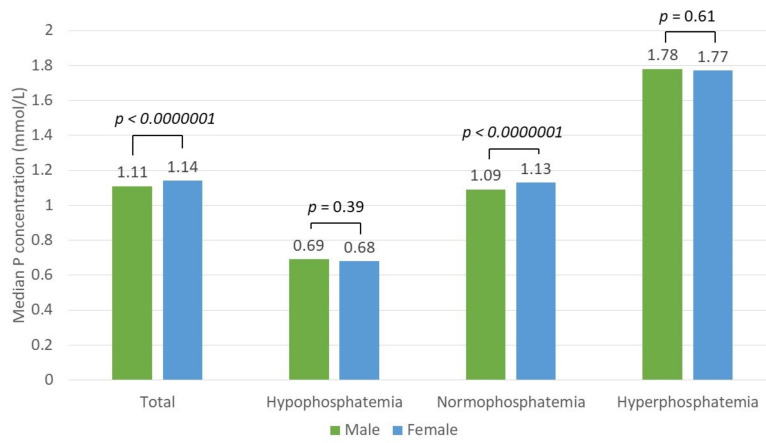
Median phosphate (P) concentration difference between sexes in the total patient and hypo-, normo-, and hyperphosphatemia groups.

There was a statistically significant difference in phosphate concentration between sexes in both the total patient (*p* < 0.0000001) and normophosphatemia groups (*p* < 0.0000001) but not in the hypophosphatemia (*p* = 0.39) or hyperphosphatemia groups (*p* = 0.61, Mann–Whitney U test) (Figure 4).

The correlation between age and serum phosphate concentration was statistically significant, but very weak (*p* < 0.05, *r* = 0.02). However, the median age differed significantly between the hypo-, normo-, and hypermagnesemia groups (*p* < 0.0001). Furthermore, there were some statistically significant differences in the median phosphate concentrations between the age groups (*p* < 0.05) (Table 4).

### 3.4. Vitamin D

Vitamin D concentration was tested in 7956 patients, among whom 2944 (37%) were men and 5012 (63%) were women. The mean vitamin D concentration was 33 ± 23.5 ng/mL. In accordance with the variable cutoff values for the lower normal limit, analysis was performed in three different settings, where 30–50 ng/mL, 20–50 ng/mL, and 10–50 ng/mL were taken as reference values, respectively.

### 3.5. Reference Values of 30–50 ng/mL

**Figure 5:**
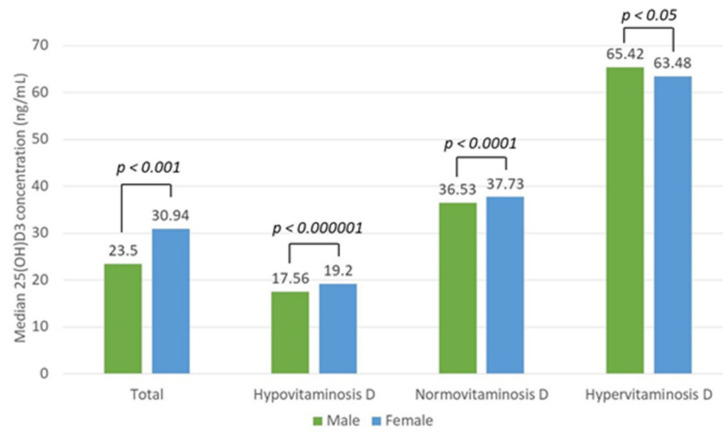
Median vitamin D (25(OH)D3) concentration difference between sexes in the total patient and hypo-, normo-, and hypervitaminosis D groups with the reference values of 30–50 ng/mL.

Hypovitaminosis D (25(OH)D3 concentration < 30 ng/mL) was found in 4296 (54%) subjects, of whom 1933 (45%) were men and 2363 (55%) were women. The patients with hypovitaminosis D had a mean vitamin D concentration of 18.1 ± 7.14 ng/mL. Normovitaminosis D (25(OH)D3 concentration of 30–50 ng/mL) was found in 2387 (30%) patients, among whom 716 (30%) were men and 1671 (70%) were women. The mean vitamin D concentration in the group with the normal vitamin D concentration was 38.1 ± 5.6 ng/mL. Hypervitaminosis D (25(OH)D3 concentration > 50 ng/mL) was found in 1273 (16%) subjects, of whom 318 (25%) were men and 955 (75%) were women. Normo- and hypervitaminosis D were significantly more frequent in women than in men (*p* < 0.0001). The patients with hypervitaminosis D had a mean vitamin D concentration of 72.7 ± 28.5 ng/mL. The median vitamin D concentration differed significantly between the three groups (*p* < 0.0001, Mood’s median test). There was a statistically significant difference in vitamin D concentration between sexes among both the total patient (*p* < 0.001) and the hypo-, hyper-, and normovitaminosis D groups (*p* < 0.000001, *p* < 0.05, *p* < 0.0001 (Mann–Whitney U test), respectively) (Figure 5).

### 3.6. Reference Values of 20–50 ng/mL

Hypovitaminosis D (25(OH)D3 concentration < 20 ng/mL) was found in 2387 (30%) subjects, of whom 1146 (48%) were men and 1241 (52%) were women. The patients with hypovitaminosis D had a mean vitamin D concentration of 12.75 ± 4.36 ng/mL.

Normovitaminosis D (25(OH)D3 concentration of 20–50 ng/mL) was found in 4296 (54%) patients, among whom 1504 (35%) were men and 2792 (65%) were women. The mean vitamin D concentration in the group with normal vitamin D concentration was 31.2 ± 8 ng/mL.

Hypervitaminosis D (25(OH)D3 concentration > 50 ng/mL) was found in 1273 (16%) subjects, of whom 318 (25%) were men and 955 (75%) were women. Normo- and hypervitaminosis D were significantly more frequent in women than in men (*p* < 0.0001). The patients with hypervitaminosis D had a mean vitamin D concentration of 72.7 ± 28.5 ng/mL.

The median vitamin D concentration differed significantly between the three groups (*p* < 0.0001, Mood’s median test).

**Figure 6:**
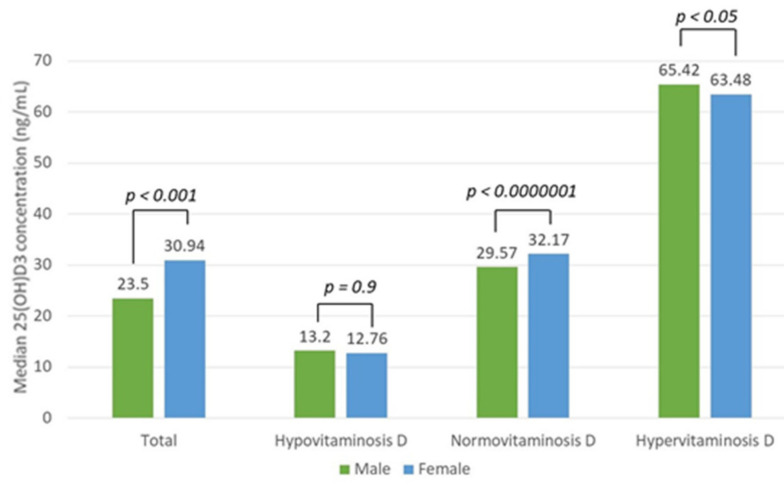
Median vitamin D (25(OH)D3) concentration difference between sexes in the total patient and hypo-, normo-, and hypervitaminosis D groups with the reference values of 20–50 ng/mL.

There was a statistically significant difference in vitamin D concentration between sexes among the total patient (*p* < 0.001) and the hyper- and normovitaminosis D groups (*p* < 0.05, *p* < 0.0000001, respectively), but not in the hypovitaminosis D group (*p* = 0.09, Mann–Whitney U test) (Figure 6).

### 3.7. Reference Values of 10–50 ng/mL

Hypovitaminosis D (25(OH)D3 concentration < 10 ng/mL) was found in 716 (9%) subjects, of whom 329 (46%) were men and 387 (54%) were women. The patients with hypovitaminosis D had a mean vitamin D concentration of 7.3 ± 1.8 ng/mL. Normovitaminosis D (25(OH)D3 concentration of 10–50 ng/mL) was found in 5967 (75%) patients, among whom 2327 (39%) were men and 3640 (61%) were women. The mean vitamin D concentration in the group with the normal vitamin D concentration was 27.4 ± 10.4 ng/mL.

**Figure 7:**
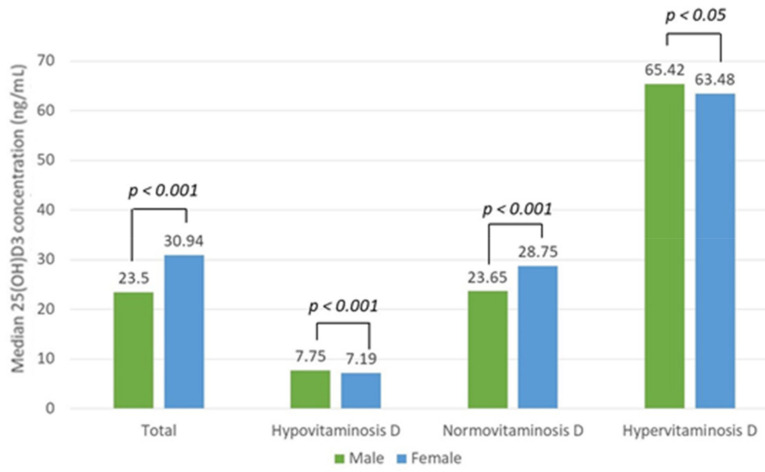
Median vitamin D (25(OH)D3) concentration difference between sexes in the total patient and hypo-, normo-, and hypervitaminosis D groups with the reference values of 10–50 ng/mL.

Hypervitaminosis D (25(OH)D3 concentration > 50 ng/mL) was found in 1273 (16%) subjects, of whom 318 (25%) were men and 955 (75%) were women. Normo- and hypervitaminosis D were significantly more frequent in women than in men (*p* < 0.0001). The patients with hypervitaminosis D had a mean vitamin D concentration of 72.7 ± 28.5 ng/mL. The median vitamin D concentration differed significantly between the three groups (*p* < 0.0001, Mood’s median test). There was a statistically significant difference in vitamin D concentration between sexes among the total patient (*p* < 0.001) and hypo-, hyper-, and normovitaminosis D groups (*p* < 0.001, *p* < 0.05, *p* < 0.001 (Mann–Whitney U test), respectively) (Figure 7).

The correlation between age and the serum vitamin D concentration was statistically significant, but very weak (*p* < 0.05, *r* = −0.03). The median age difference between the hypo-, normo-, and hypervitaminosis D groups depended on the adopted reference values. There was no statistically significant difference when the reference values of 30–50 ng/mL were adopted (*p* = 0.35). However, there were statistically significant differences between all the groups (*p* < 0.05) when the reference values of 20–50 ng/mL were used as well as when the reference values of 10–50 ng/mL were adopted (*p* < 0.0001, Kruskal–Wallis test). Additionally, there were some statistically significant differences in the median vitamin D concentrations between the age groups (*p* < 0.00001) (Table 5).

### 3.8. Vitamin D and Calcium

Both vitamin D and calcium concentrations were tested in 4107 patients, among whom 1643 (40%) were men and 2464 (60%) were women. The mean vitamin D concentration was 31 ± 20.3 ng/mL, and the mean calcium concentration was 2.4 ± 0.21 mmol/L. There was no statistically significant correlation between the serum vitamin D and calcium concentration (*p* = 0.15, *r* = 0.02). Furthermore, there was no statistically significant difference in the median vitamin D concentration between the hypo-, normo-, and hypercalcemia groups (*p* = 0.27), and neither did the median calcium concentration differ between the hypo-, normo-, and hypervitaminosis D groups, independent of the adopted reference values of 30–50 ng/mL, 20–50 ng/mL, or 10–50 ng/mL (*p* = 0.38, *p* = 0.22, *p* = 0.15 (Kruskal-Wallis test), respectively).

## 4. Discussion

Calcium–magnesium–phosphate homeostasis disorders are still an incompletely studied phenomenon, and the results regarding their prevalence are not consistent. In our study, dyscalcemia was found in 23.5% of the serum calcium test results, dysmagnesemia—in 33% of the serum magnesium test results, dysphosphatemia—in 26% of the serum phosphate test results, and dysvitaminosis D (depending on the adopted reference values)—in 70% (30–50 ng/mL), 46% (20–50 ng/mL), or 25% (10–50 ng/mL) of the serum vitamin D test results. These values are lower than those reported by other authors [15,16,17,18,19,20,21,22]. This may be caused by the fact that we analyzed the general population of hospitalized patients instead of selecting those with calcium, magnesium, or phosphate homeostasis disturbances as a primary cause of hospitalization. We need to underline that in the enrolled study group, there were people who were hospitalized due to different causes; some of them could influence the calcium–magnesium–phosphorus balance; however, the participation of patients from particular departments was equal (only the Hematology Department and the Oncology Department constituted 20% of the enrolled subjects). Nevertheless, since even mild abnormalities of calcium, magnesium, phosphates, and vitamin D have negative health effects and may increase the risk of death, these findings are alarming [4].

In our study, hypocalcemia was present in 18.5% of the patients undergoing serum calcium testing. The reported prevalence of hypocalcemia is higher and ranges from 30% to 85% of patients, depending on the study group [16,17,23,24]. The disorder is most commonly seen in the intensive care unit, and the elderly are at greater risk of its development, but only 4% of our patients were from this department, which may explain the lower percentage of patients with this disorder in our study [15,16].

Hypercalcemia was found in 5% of the patients undergoing serum calcium testing, which is in line with the literature data [24]. However, in our study, hypercalcemia was significantly more common in women (*p* < 0.0001). This may be explained by the fact that primary hyperparathyroidism is most commonly found in postmenopausal women, which in turn is the main cause of hypercalcemia [25]. This condition can also be caused by medications, granulomatous disorders, and cancer [26]. Another possibility is the influence of hypervitaminosis D, which was significantly more frequent in women in our study group (*p* < 0.0001), which is also in line with the results of Dudenkov et al. [27].

Interestingly, we did not find any statistically significant correlation between calcium and vitamin D concentrations, whereas the literature describes the phenomenon of a more frequent occurrence of hypocalcemia in persons with abnormal vitamin D concentrations as vitamin D influences the gut absorption of calcium [28,29]. This may be due to the fact that reduced intestinal absorption may be masked by the release of calcium from the skeleton and thus not affect serum calcium levels for a long time.

The results for vitamin D deficiency in the study group depend on the cutoff point used. When the optimal vitamin D concentration cutoff point is 30 ng/mL, as many scientists have proposed, 54% of the study group is vitamin D-deficient [30]. This result is consistent with those obtained by Shea et al. [31]. However, some authors report a much higher prevalence of hypovitaminosis D, even at 93.8% of elderly patients [22]. The discrepancy in the results (54% vs. 93.8%) may be due to the selection of the study group.

On the other hand, if lower cutoff points are taken, hypovitaminosis D affects 30% of the group when 20 ng/mL and above is considered normal and 9% of the group when 10 ng/mL is considered normal. Some authors suggest that lower vitamin D concentrations are sufficient because higher scores do not improve patient’s health [32]. The prevalence of vitamin D deficiency in only 30% of the study group is much lower than that estimated by the Endocrine Society, which places the prevalence of vitamin D deficiency at 80–100% of older people in Europe and the United States [33]. Our result of 9% of patients with vitamin D levels below 10 ng/mL is similar to the values described by the European Calcified Tissue Society [34].

The prevalence of hypervitaminosis D found in our study was not significantly higher than that reported by Dudenkov et al., 16% vs. 9.3% [27]. Hypervitaminosis D is uncommon given the consumption of natural sources of this vitamin, as well as its synthesis through sunlight exposure. Therefore, such a high percentage of hypervitaminosis D cases may probably have been caused by excessive intake of this vitamin by patients in the form of dietary supplements, which is sometimes caused by poor manufacturing practices [35,36].

In our study, the prevalence of hypomagnesemia was similar to the results of Cheungpasitporn et al., 26% vs. 20.2% [21]. Hypomagnesemia is very unfavorable in hospitalized patients because it increases the risk of in-hospital death, sepsis, the need for mechanical ventilation, and prolonged hospital stay [37]. Hypomagnesemia is caused by a number of factors, including vomiting, diarrhea, chronic parenteral fluid therapy, hypercalcemia, and medications [38]. Hypomagnesemia is more common in the elderly not only because of the medications they take, but also because of a deficient diet [39].

The prevalence of hypermagnesemia found in this study is similar to the values described by other investigators, 7% vs. 12% [21]. Although hypermagnesemia is still described as a rare phenomenon in people without renal failure [40], this condition is more common in elderly patients, especially those burdened with severe comorbidities, so its prevalence may have been relatively high in the group of hospitalized patients older than 65 years [18].

The prevalence of hypophosphatemia among hospitalized patients reported in the literature is between 20% and 40% [41]. The results obtained in our study are lower: 11%. Hypophosphatemia is particularly common in patients with certain medical conditions, such as ketoacidosis, sepsis, and after heart and liver surgery [42]. Since data on the reason for patients’ hospitalization were not included in the analysis, we cannot address whether the study group had a particularly small number of patients with such conditions. On the other hand, hypophosphatemia is rarely diagnosed because it does not produce specific symptoms in patients other than fatigue and increased irritability [43].

The prevalence of hyperphosphatemia in our study was high compared with the results from another study, 15% vs. 0.5%, respectively [44]. The difference could be caused by the selection of the study group. In our study, we analyzed the results of the general hospital population, whereas Tonelli et al. analyzed the results of a group of patients with coronary heart disease [44].

We found almost no effect of age on the calcium and phosphate levels, perhaps because all the patients in our study group were aged 65 years and over. In the case of magnesium, our results are in line with those of other authors, who found that magnesium concentrations are age-dependent, and older people are more likely to have abnormal magnesium concentrations [21,39]. For vitamin D, both in our study and in the studies of other authors, there was a correlation with age [27].

In our study, sex had a statistically significant effect on the serum calcium, magnesium, and vitamin D concentrations. In the case of calcium concentration, the greatest association with sex is with the presence of hypocalcemia, which is more commonly found in males. In contrast, the results regarding hypercalcemia are not consistent [16,24]. The results of other investigators regarding the sex dependence of magnesium concentrations are, again, partly in line with ours, and hypomagnesemia is found more often in women, while there is no correlation in the group with hypermagnesemia [39]. Phosphate concentrations, on the other hand, do not seem to be sex-dependent at all, which is also partly evident in our results [45]. Vitamin D concentration appears to be sex-dependent, as in our study, normo- and hypervitaminosis D were significantly more common in women irrespective of the adopted reference values, similarly to the results of Dudenkov et al. [27].

Our study has some strengths, which are worth mentioning. Firstly, a large sample size (66,450 results including 4107 calcium results, 31,690 magnesium results, 22,707 phosphates results, and 7956 vitamin D3 results) significantly reduces the risk of randomness of the obtained results. Secondly, there are few current studies assessing the incidence of abnormal concentrations of the investigated minerals, especially of phosphates, in the hospital population. The increase in the frequency of observed hypermagnesemia over the years indicates the need for cyclical conduct of this type of research.

However, it also has limitations that should be stated. First, this study is based on evaluation of the results (raw data) which we had access to in the laboratory information system, not the patients and their clinical presentation. To avoid errors in the analysis as much as possible, we eliminated repeated measurements performed for the same patients. Second, we did not eliminate the patients who might have been taking medications that could affect the results. We only estimated that they constituted 12.5% of the studied group. This estimation was based on the exact analysis of 1000 randomly selected medical reports of the included subjects. All the extracted results were initially set in chronological order (according to the date of analysis), and every 50th result was taken for medical report analysis. We decided not to eliminate these results from the final analysis because we suspected that the data regarding drug administration might have been incomplete and underestimated. There were no data on the doses of these drugs, their duration of use and information on other preparations and supplements that could have been taken by the patients, which is not uncommon in the elderly group, and which could also affect the concentrations of the parameters tested. Thus, including all the obtained results seemed to be the most appropriate approach. Third, the method of vitamin D concentration measurement that was available to us was only an immunoassay. This is not the gold standard (which is mass spectrometric methods); therefore, the results bear a risk of inaccuracy. Fourth, calcium homeostasis is difficult to assess, and even if the measurement is corrected for albumin in patients with hypoalbuminemia, there is a risk of obtaining incorrect results. Finally, in this study, calcemia, magnesemia, phosphatemia, and D3 vitaminosis are analyzed independently. We included the results of the patients who had at least one of the parameters tested, not all four at the same time. The reason is that physicians very often order only one or two of these parameters to be tested. If we were to extract only the patients with all the four determined parameters, the number of included patients would be considerably lower. Nevertheless, such an analysis would be of high interest and, after collecting the representative number of results, we will promptly share our findings.

## 5. Conclusions

In conclusion, disorders of the calcium–magnesium–phosphate metabolism are common in hospitalized patients over 65 years of age with dysmagnesemia as the most common disturbance. These disorders have a negative impact on health and are associated with an increased risk of death. Since the elderly, due to the burden of comorbidities, long-term medication use, and deficient diet, are more prone to calcium–magnesium–phosphate homeostasis disturbances, the concentrations of these substances should be routinely monitored in this group.

## Figures and Tables

**Table 1:** Percentage of patients from individual hospital departments.

Departments	Percentage of Patients (%)
Diabetology	6.10
Cardiology	5.20
General Surgery	2.92
Endocrinology	4.59
Hematology and Oncology	21.12
Nephrology	4.65
Neurology	4.65
Laryngology	1.20
Intensive Care	4.00
Gastroenterological Surgery	7.53
Vascular Surgery	3.59
Hepatic Surgery	7.13
Neurosurgery	2.05
Cardiosurgery	1.31
Hypertensiology and Internal Medicine	3.36
Gastroenterology	5.11
Pneumonology	2.73
Nephrology and Dialysis	5.43
Hepatology	7.34

**Table 2:** Basic information regarding the study group.

	Calcium	Magnesium	Phosphates	Vitamin D
Number of patients	4107	31,680	22,707	7956
Sex	40% male	52% male	52% male	37% male
Age (years), mean ± SD	74.76 ± 7.6	75.0 ± 7.68	75 ± 7.4	74.4 ± 7.9
Mean Concentration ± SD	2.4 ± 0.2 mmol/L	0.82 ± 0.14 mmol/L	1.19 ± 0.4 mmol/L	33 ± 23.5 ng/ml
Median	2.39 mmol/L	0.82 mmol/L	1.125 mmol/L	28.2 ng/ml
Minimum–maximum	1.56–4.33 mmol/L	0.14–3.14 mmol/L	0.11–6.04 mmol/L	3.03–149.54 ng/ml
Hypoconcentration	18.5%	26%	11%	54%
Hyperconcentration	5%	7%	15%	16%

**Table 3:** Comparison of the median calcium concentrations between the age groups (Kruskal–Wallis test).

	Median Calcium Concentration	*p*
Age group, 64–74	2.400	<0.05
Age group, 75–84	2.379	
Age group, 65–74	2.400	0.3
Age group, 85–94	2.390	
Age group, 65–74	2.400	1
Age group, ≥95	2.420	
Age group, 75–84	2.379	1
Age group, 85–94	2.390	
Age group, 75–84	2.379	1
Age group, ≥95	2.420	
Age group, 85–94	2.390	1
Age group, ≥95	2.420	

**Table 4:** Comparison of the median phosphate concentration between the age groups (Kruskal–Wallis test).

	Median Phosphate Concentration	*p*
Age group, 64–74	1.18	1
Age group, 75–84	1.19	
Age group, 65–74	1.18	<0.05
Age group, 85–94	1.2	
Age group, 65–74	1.18	1
Age group, ≥95	1.2	
Age group, 75–84	1.19	<0.05
Age group, 85–94	1.2	
Age group, 75–84	1.19	1
Age group, ≥95	1.2	
Age group, 85–94	1.2	1
Age group, ≥95	1.2	

**Table 5:** Comparison of the median vitamin D concentration between the age groups (Kruskal–Wallis test).

	Median Vitamin D Concentration	*p*
Age group, 64–74	32.9	0.4
Age group, 75–84	34.0	
Age group, 65–74	32.9	<0.00001
Age group, 85–94	31.0	
Age group, 65–74	32.9	<0.00001
Age group, ≥95	22.4	
Age group, 75–84	34.0	<0.000001
Age group, 85–94	31.0	
Age group, 75–84	34.0	<0.00001
Age group, ≥95	22.4	
Age group, 85–94	31.0	<0.001
Age group, ≥95	22.4	

## Data Availability

The data presented in this study are available upon request from the corresponding author. The data are not publicly available due to ethical reasons.

## Abbreviations

Ca, calcium; Mg, magnesium; P, phosphate; 25(OH)D3, vitamin D.
